# Challenges in sustaining the elimination of visceral leishmaniasis in India

**DOI:** 10.1093/inthealth/ihaf063

**Published:** 2025-06-18

**Authors:** Mitali Chatterjee, Syamal Roy, Simon L Croft

**Affiliations:** Dept. of Pharmacology, Institute of Postgraduate Medical Education & Research, 244B AJC Bose Road, Kolkata 700 020, India; Indian Association for the Cultivation of Science, Kolkata 700032, India; London School of Hygiene and Tropical Medicine, WC1E 7HT, UK

## Abstract

The South East Asian initiative for elimination of kala-azar from the Indian subcontinent that began in 2005 is coming to fruition, with India in the last mile of elimination. This aptly timed commentary based on the publication of Pandey et al. (2025) entitled ‘Kala-azar elimination in India: reflections on success and sustainability’ highlights the complementarity of political commitment that ensured socioeconomic development, along with evidence-based operational research, that needs to be sustained for zero transmission to become a reality.

Twenty years ago, in Geneva, the governments of Bangladesh, India and Nepal committed to an initiative to eliminate visceral leishmaniasis (VL or kala-azar) as a public health problem from the region (https://iris.who.int/handle/10665/205825) by setting the target at reducing the disease incidence to less than one VL case per 10 000 population at the subdistrict or district level per year. The review published in *International Health*^1^ on progress towards this goal in India is well timed, being based upon recent assessments made by WHO-led teams. The tenets for elimination in 2005 in South East Asia were early diagnosis (based upon the then recently developed field adapted dipstick rK39) and complete treatment with miltefosine, the first oral drug for VL, coupled to knowledge that the only vector was *Phlebotomus argentipes* (pyrethroid susceptible) and that there was no animal reservoir (i.e. an anthroponotic disease). Twenty years later, rK39 continues to be the key diagnostic tool but the drug of choice is now a single infusion of liposomal amphotericin B, AmBisome. The programme continues to focus on vector control and improved surveillance to reduce transmission. Active case surveillance proved a game changer, in that Post kala-azar Dermal Leishmaniasis (PKDL) cases with ‘macular and hypopigmented lesions’ were identified, which owing to their relatively innocuous features had remained below the radar. However, advances in vector control have lagged behind considerably. Importantly, perspectives for, and confidence in the elimination of VL, have been augmented with: (i) the introduction in 2011 of a single course, AmBisome; (ii) the use of drug combinations in HIV-VL cases; and (iii) xenodiagnoses-based quantifiable evidence that confirmed PKDL cases are the disease reservoir, and the strongest contender to sustain transmission.

The review of progress by Pandey et al.^1^ towards elimination of VL is comprehensive and has a strong flavour of optimism as the case numbers have dramatically declined within the endemic countries and South Asia is now contributing a mere 6% of the world's VL cases. Bangladesh in 2016 and Nepal in 2023 have achieved the WHO-prescribed elimination target, although the emergence of new foci, especially of cutaneous leishmaniasis in Nepal with the causative species being *Leishmania donovani*, raises alarm bells.^2^

It is important now to reflect further on this target as: (i) the definition of elimination has been changed in the recent WHO NTD Roadmap (https://www.who.int/publications/i/item/9789240010352); and (ii) strategies need modification as countries move from elimination as a public health problem to eradication and zero transmission. In this respect, there are additional germane points to consider, for example:

- Unlike viral diseases, such as smallpox and polio, with their successful vaccination programmes, there is no vaccine for VL or PKDL, and unlikely to be one for at least a decade. More research and development is needed to identify potential susceptibility factors that cause a small population (2–10%) of VL cases to develop PKDL.
- AmBisome, which played a critical role in elimination, has been donated free for treatment of VL by the manufacturing pharmaceutical company via the WHO. Economic and political factors need to be addressed and governments prepared to pay the full cost. Another challenge is the absence of an alternative drug regimen to replace the 12-wk treatment with miltefosine for PKDL, which has the potential for developing ocular complications and is fraught with non-compliance.
- VL has been a ‘mobile’ disease as seen in India over the past two centuries. New foci of VL can arise in unexpected regions, as reported in Nepal and Bangladesh. Atypical cutaneous leishmaniasis caused by *L. donovani* is on the rise in Sri Lanka, India and Nepal, and can potentially fuel future VL outbreaks and derail the elimination programme.^2,3^ In another study in Bihar, India, both in outbreak and in endemic villages, *L. donovani* was detectable in dogs by qPCR through xenodiagnosis using laboratory-grown *P. argentipes.*^4^ In summary, appropriate tools for surveillance, a population diagnostic not just a patient diagnostic, need to be developed and integrated into health system surveillance and monitoring programmes coupled with community awareness. There is also a need to develop mapping systems that can monitor changes in the biology and distribution of the vector, especially with the looming threat of climate change.
- To ignore the possibility of a non-human mammalian reservoir could also have a long-term adverse impact; there remains a need for further research on potential reservoirs. Dogs are a potential reservoir in India as *L. donovani* parasites have been identified in peripheral blood samples from domestic dogs (n=47) and wild rats (n=25), although a definitive demonstration of Leishman Donovan bodies in splenic and bone marrow aspirates of dogs was not established.^4,5^
- Conventionally, in the Indian subcontinent, *P. argentipes* is considered as the vector for disease transmission. However, nationwide entomological surveillance to detect *L. donovani* in phlebotomine sand flies in five different biogeographical zones in India established that, in addition to *P. argentipes*, which is prevalent in Bihar, West Bengal and Kerala, *Phlebotomus sergenti, Phlebotomus papatasi, Phlebotomus longiductus, Phlebotomus major* and *Phlebotomus bruneyi* were detected, suggesting that rigorous vector surveillance should be incorporated into the National Kala-Azar Elimination Program.^6^
- Finally, the 2005 agreement was regional, not single-country based. This review has also to be seen within the context of the WHO validation criteria for elimination (WHO, 2016) progress made in Nepal^7^ and the remarkable achievement of elimination in Bangladesh,^8^ where now the target is zero transmission. The mobility of humans across borders, the contiguity of vector populations without geographical boundaries, make it essential that all measures to sustain elimination and move towards zero elimination are considered as a regional solution.

Lessons also need to be learned from other infectious disease programmes, not least within the Malaria Eradication Programme. The MalERA Refresh document^9^ details steps required and should be compulsory reading for all those who are considering the needs for further research and development and further funding. The premature declaration of leprosy elimination in 2005 led to lowered activity in the control and research programmes, unabated transmission, an absence of new improved treatments and patient care, along with few thoughts on sustainability, leading to the waves of resurgence remaining unnoticed as a function of time. Experts argue whether this was a ‘forced elimination’ that diminished the country's ability to tackle the disease in a better way.^10^ Following a new focus on research, Leprosy Elimination Monitoring Tools are being implemented (https://www.who.int/publications/i/item/9789290210474), understanding of epidemiology has been improved and new drugs are close to clinical trials. The question still remains whether, as in leprosy, we may face a similar rhetoric in the ongoing VL elimination programme in the Indian subcontinent. The article by Pandey et al.^1^ lists the required next steps in the section ‘Considerations for the future—what next?’. The bottom line for VL elimination leading to zero transmission is sustained research, sustained funding and sustained engagement of health services and communities.

## Data Availability

None.
